# Outpatient visit trends for internal medicine ambulatory care sensitive conditions after the COVID-19 pandemic: a time-series analysis

**DOI:** 10.1186/s12913-022-07566-6

**Published:** 2022-02-14

**Authors:** Ciara Pendrith, Dhruv Nayyar, Cherry Chu, Tara O’Brien, Owen D. Lyons, Payal Agarwal, Danielle Martin, R. Sacha Bhatia, Geetha Mukerji

**Affiliations:** 1 Temerty Faculty of Medicine, University of Toronto, Toronto, ON Canada; 2 Women’s College Hospital Institute for Health System Solutions & Virtual Care, 76 Grenville Street, Toronto, Ontario M5S 1B3 Canada; 3 Institute of Health Policy, Management and Evaluation, Dalla Lana School of Public Health, University of Toronto, Toronto, ON Canada; 4 Women’s College Hospital, Toronto, ON Canada; 5 Department of Family and Community Medicine, University of Toronto, Toronto, ON Canada; 6 Peter Munk Cardiac Centre, University Health Network, Toronto, ON Canada

**Keywords:** Virtual care, COVID-19 pandemic, Internal medicine, Ambulatory care sensitive conditions, Health services

## Abstract

**Background:**

The COVID-19 pandemic led to a dramatic shift in the delivery of outpatient medicine with reduced in-person visits and a transition to predominantly virtual visits. We sought to understand trends in visit patterns for ambulatory care sensitive conditions (ACSCs) commonly seen in internal medicine clinics.

**Methods:**

We included adult outpatients seen for an ACSC between March 15th, 2017 and March 14th, 2021 at a single-centre in Ontario, Canada. Monthly visits were assessed by visit type (new consultation, follow-up), diagnosis, and clinic. Time series analyses compared visit volumes pre- and post-pandemic. Proportion of virtual visits were compared before and during the pandemic. Patient and visit factors were compared between in-person and virtual visits.

**Results:**

8274 patients with 34,021 visits were included. Monthly visits increased by 15% during the pandemic (*p* <  0.0001). New consultations decreased by 10% (*p* = 0.0053) but follow-up visits increased by 21% (p <  0.0001). Monthly heart failure visits increased by 43% (*p* <  0.0001) whereas atrial fibrillation visits decreased. Pre- pandemic, < 1% of visits were virtual compared to 82% during the pandemic (p <  0.0001). Less than half of heart failure visits were virtual whereas > 95% of diabetes visits were virtual.

**Conclusions:**

We found a significant increase in overall visits to internal medicine clinics driven by increased volumes of follow-up visits, which more than offset decreased new consultations. There was variability in visit trends and uptake of virtual care by visit diagnosis, which may indicate challenges with delivery of virtual care for certain conditions.

## Background

The COVID-19 pandemic led to rapid changes in the delivery of healthcare services to meet patient needs throughout the pandemic while reducing the risk of exposure to SARS-CoV-2. Among other adaptations, healthcare systems rapidly transitioned from traditional in-person office-based visits to predominantly virtual care [1–4].

Within Ontario, a Canadian province with a population of 14,500,000 and universal health insurance, adoption of virtual care was low before the pandemic [5, 6]. Barriers included challenges with reimbursement, policy issues, and limited access to technology [1, 7–9]. Within Ontario specifically, virtual delivery of physician services were previously only covered by the Ontario Health Insurance Plan if delivered from a government-approved facility using a government-approved telemedicine platform [3, 5].^3,5^ After the declaration of COVID-19 as a global pandemic, new temporary billing codes were issued on March 14th, 2020, which allowed physicians to be renumerated for outpatient visits conducted via telephone or videoconference at the same rate as an in-person visit which facilitated the widespread adoption of virtual care [3, 10]. While previous studies have examined changes in visit volumes and uptake of virtual care during the pandemic, these have generally been limited to primary care [4, 5, 10] or narrow scopes of care [11, 12].

To our knowledge, no study to date has assessed the COVID-19 pandemic’s impact on visit trends and utilization of virtual care across multiple internal medicine ambulatory care sensitive conditions (ACSCs). ACSCs are health conditions where high quality and timely care may prevent acute exacerbation, sequelae of disease, and emergency department visits or hospitalizations [13]. By assessing changes in outpatient visits for several ACSCs across a single institution, we can understand the relative impact of the pandemic and virtual care on different conditions. These findings may highlight areas where virtual care is better suited than others. Additionally, understanding care patterns for multiple ACSCs may help organizations plan future allocation of resources within an evolving healthcare environment in the ongoing pandemic with the goals of preventing clinical deterioration and avoidable inpatient visits.

Our objectives were to understand how the onset of the COVID-19 pandemic and introduction of virtual care billing codes impacted patterns of care and uptake of virtual care for outpatients with common internal medicine ACSCs at a large academic ambulatory hospital in Ontario, Canada. Furthermore, we sought to understand patient and visit factors associated with utilization of virtual care.

## Methods

### Design, setting and participants

We conducted a repeated cross-sectional study of all patients seen in internal medicine clinics for an ACSC at Women’s College Hospital (WCH) in Toronto, Ontario between September 15th, 2017 and March 14th, 2021. WCH is an ambulatory care hospital affiliated with the University of Toronto.

Internal medicine clinics included were the following: General Internal Medicine (GIM) clinic; Acute Ambulatory Care Unit, which is a short stay (< 24 h) unit providing urgent assessment, and treatment of internal medicine conditions; Cardiology clinic; Atrial Fibrillation clinic; General Endocrinology clinic; Diabetes clinic; and Respirology clinic. ACSCs included in this study were hypertension, congestive heart failure, coronary artery disease, atrial fibrillation, diabetes mellitus, asthma, and chronic obstructive pulmonary disease [13].

Patient visits meeting the following criteria were included: 1) main visit diagnosis was an ACSC to an internal medicine clinic; and 2) visit occurred during the study time period. Visits were included if the healthcare provider was a physician, nurse practitioner, or nurse. Both new consultations and follow-up visits were included. Patient encounters were excluded if the encounter was for a procedure with no corresponding office or virtual care visit. Multiple same day visits to different providers were excluded if the encounter ID was the same (e.g. seen by physician and nurse for the same encounter).

The study period is divided into pre-pandemic and pandemic periods which are defined as September 15th, 2017-March 14th, 2020 and March 15th, 2020-March 14th, 2021, respectively. The study period was selected to include two and a half years of data before and one year after the introduction of temporary virtual care billing codes on March 14th, 2020 to allow sufficient data points to adjust for pre-pandemic trends in the time series analysis.

### Data sources

Data for this study were collected from the hospital’s electronic health record EPIC. Data on visit information and patient characteristics were extracted electronically using a data query, and a manual chart review was performed for any missing or unknown variables. Video visits were delivered through Zoom videoconferencing technology [14] integrated through the electronic medical record patient portal myHealthRecord [15].

### Baseline characteristics

For each included visit we extracted the visit date, main diagnosis using the diagnostic code, clinic, visit type (new consultation, follow-up visit), and visit modality (in-person, or virtual including telephone and videoconference visits). The following baseline patient characteristics were captured at the time of each visit: age, sex, and whether the patient had an email registered.

### Outcomes

Our primary outcome was change in average monthly visits after the onset of the pandemic and introduction of virtual care billing codes. We assessed changes in visit volumes overall, as well as by visit type to understand changes in number of new consultations and follow-up visits. Visit volumes by visit diagnoses were compared to understand differences in the types of patient conditions being seen. Additionally, we assessed the proportion of virtual visits in the pre-pandemic and pandemic periods. Secondary outcomes included patient and visit factors associated with virtual care vs. in-person visits in the pandemic period.

### Statistical analyses

Baseline characteristics of patients and visits were compared before and during the pandemic using Chi-square tests and t-tests. Average monthly visit numbers were compared during the pre-pandemic and pandemic periods using two-sample t-tests and the change in monthly visits was calculated as both the mean and relative difference. To understand differences in pandemic visit volumes compared with historical data, time series analyses were performed using interventional Autoregressive Integrated Moving Average (ARIMA) models to examine the statistical significance of the change in visit volumes from pre-pandemic to during. Models were run for total visits, new consultations, follow-ups, and each visit diagnosis. Interventional ARIMA modelling is commonly used to analyze the effect of change (e.g. major event or intervention) in a time-series analysis [16]. A step function was applied to the model to characterize the start of the pandemic. Secondary outcomes of factors associated with virtual visits in the pandemic period were assessed using Chi-square tests and two-sample t-tests. *P*-values < 0.05 were considered statistically significant. All statistical analyses were completed using Microsoft Excel and SAS version 9.4.

### Ethics

Ethics approval was received from the Research Ethics Board at Women’s College Hospital (REB approval #: 2019–0191-E).

## Results

### Study participants and visits

A total of 8274 unique patients with 34,021 outpatient visits were included in the study. Most patients had multiple visits during the study time period, with 28.2% having one visit, 16.9% having two visits, and 54.9% having three or more visits. Characteristics of patients and their visits are included in Table 1. The mean age was 58.1 years and 61.4% of visits were delivered to female patients; 52.3% of patients had an email registered. There were no differences in patient demographics in the pre-pandemic and pandemic periods.

**Table 1:** Baseline characteristics of patients and visits in the pre-pandemic and pandemic periods

	Pre-pandemic *n* = 23,279	Pandemic *n* = 10,742	Overall *n* = 34,021	***p***-value
*Patient characteristics*				
Mean age (SD)	58.1 (18.9)	58.1 (19.0)	58.1 (18.9)	0.99
Sex				0.35
Female	14,331 (61.6%)	6556 (61.0%)	20,887 (61.4%)	
Male	8948 (38.4%)	4186 (39.0%)	13,134 (38.6%)	
Email registered	11,650 (50.0%)	6151 (57.3%)	17,801 (52.3%)	< 0.0001
*Visit characteristics*				
Visit type				< 0.0001
New consultation	4007 (17.2%)	1436 (13.4%)	5443 (16.0%)	
Follow-up	19,272 (82.8%)	9306 (86.6%)	28,578 (84.0%)	
Visit modality (grouped)				< 0.0001
In-person	23,261 (99.9%)	1943 (18.1%)	25,204 (74.1%)	
Virtual care	18 (0.1%)	8799 (81.9%)	8817 (25.9%)	
Visit modality (specified)				< 0.0001
In-person	23,261 (99.9%)	1943 (18.1%)	25,204 (74.1%)	
Telephone	18 (0.1%)	8388 (78.1%)	8406 (24.7%)	
Videoconference	0 (0.0%)	411 (3.8%)	411 (1.2%)	
Visit diagnosis				< 0.0001
AFib	1797 (7.7%)	590 (5.5%)	2387 (7.0%)	
CAD	2097 (9.0%)	898 (8.4%)	2995 (8.8%)	
CHF	1319 (5.7%)	752 (7.0%)	2071 (6.1%)	
HTN	3285 (14.1%)	1571 (14.6%)	4856 (14.3%)	
DM	10,953 (47.1%)	5088 (47.4%)	16,041 (47.2%)	
Asthma	2732 (11.7%)	1340 (12.5%)	4072 (12.0%)	
COPD	1096 (4.7%)	503 (4.7%)	1599 (4.7%)	
Clinic				< 0.0001
AACU	1758 (7.6%)	905 (8.4%)	2663 (7.8%)	
AFib clinic	663 (2.8%)	115 (1.1%)	778 (2.3%)	
Cardiology clinic	6012 (25.8%)	2768 (25.8%)	8780 (25.8%)	
GIM clinic	655 (2.8%)	248 (2.3%)	903 (2.7%)	
Endocrine clinic	8070 (34.7%)	3836 (35.7%)	11,906 (35.0%)	
DM clinic	2428 (10.4%)	1066 (9.9%)	3494 (10.3%)	
Respirology clinic	3693 (15.9%)	1804 (16.8%)	5497 (16.2%)	

*AFib* atrial fibrillation, *CAD* coronary artery disease, *CHF* congestive heart failure, *HTN* hypertension, *DM* diabetes mellitus, *COPD* chronic obstructive pulmonary disorder, *AACU* Acute Ambulatory Care Unit, *GIM* general internal medicine

There were 23,279 and 10,742 visits in the pre-pandemic and pandemic periods, respectively. The proportion of new consultation visits decreased from 17.2% pre-pandemic to 13.4% during the pandemic (*p* <  0.0001). Pre-pandemic, 99.9% of visits were conducted in-person with only 18 virtual visits, all of which were telephone visits for diabetes patients seen in the General Endocrinology and Diabetes clinics. In the pandemic period, the proportion of in-person visits decreased to 18.1% (*p* <  0.0001) with the majority of virtual visits being conducted over telephone (95.3%) and a minority by videoconference (4.7%).

### Visit trends

**Fig. 1:**
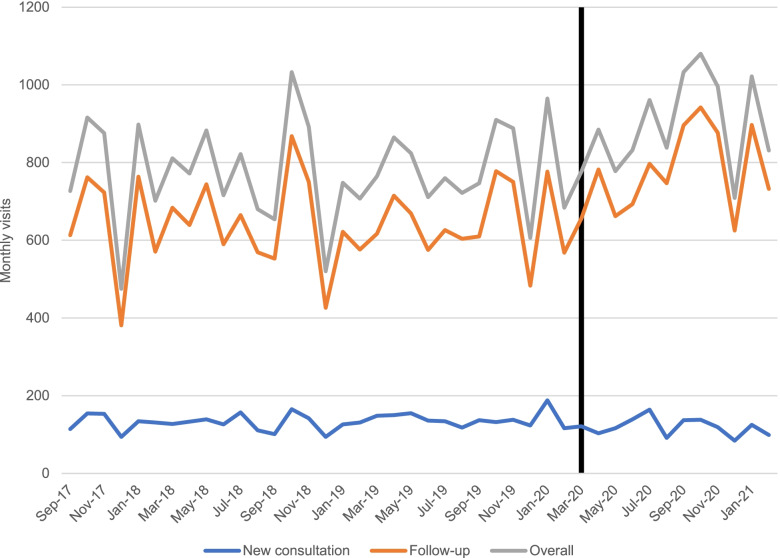
Average monthly overall, new consultation, and follow-up visits. The black vertical line represents the start of the COVID-19 pandemic

Figure 1 displays monthly overall visits, new consultations, and follow-up visits across the study period. Results from the time series analysis are shown in Table 2. Average monthly overall visits decreased by 15% between the pre-pandemic and pandemic periods (776 vs. 895), new consultations decreased by 10% (134 vs. 120), and follow-ups increased by 21% (642 vs. 776). The changes in visit volumes were significant for all types of visits: overall visits (*p* <  0.0001), new consultations (0.0053), and follow-ups (p <  0.0001).

**Table 2:** Average monthly visits in the pre-pandemic and pandemic periods

	Pre-pandemic mean visits (±SD)	Pandemic mean visits (±SD)	Mean difference	Relative difference	***p***-value*
Overall visits	776 (±126)	895 (±120)	+ 119	+ 15%	< 0.0001
New consults	134 (±21)	120 (±23)	−14	−10%	0.0053
Follow-up visits	642 (±109)	776 (±108)	+ 133	+ 21%	< 0.0001
AFib visits	60 (±14)	49 (±9)	−11	−18%	0.013
CAD visits	70 (±19)	75 (±14)	+ 5	+ 7%	0.42
CHF visits	44 (±8)	63 (±17)	+ 19	+ 43%	< 0.0001
HTN visits	110 (±20)	131 (±27)	+ 21	+ 20%	0.0049
DM visits	365 (±57)	424 (±58)	+ 59	+ 16%	< 0.0001
Asthma visits	91 (±23)	112 (±23)	+ 21	+ 23%	< 0.0001
COPD visits	37 (±10)	42 (±8)	+ 5	+ 15%	0.10

*AFib* atrial fibrillation, *CAD* coronary artery disease, *CHF* congestive heart failure, *HTN* hypertension, *DM* diabetes mellitus, *COPD* chronic obstructive pulmonary disorder

*p-values are derived from ARIMA models

**Fig. 2:**
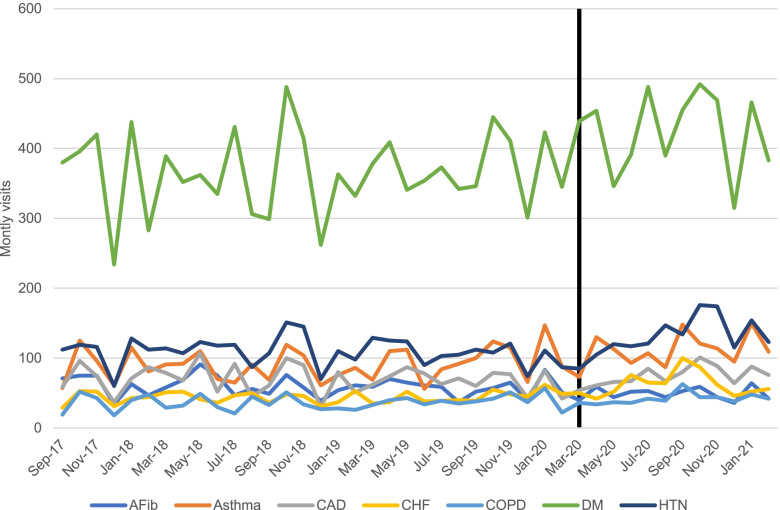
Average monthly visits by visit diagnosis. The black vertical line represents the onset of the COVID-19 pandemic. AFib = atrial fibrillation, CAD = coronary artery disease, COPD = chronic obstructive pulmonary disorder, DM = diabetes mellitus, HTN = hypertension

Average monthly visits by visit diagnosis are displayed in Fig. 2. Monthly visits for atrial fibrillation decreased by 18% (60 vs. 49) while congestive heart failure visits increased by 43% (44 vs. 63). The change in visit volumes was significant for both atrial fibrillation (*p* = 0.013) and congestive heart failure (*p* <  0.0001). There were increases in monthly visits for hypertension (20%), diabetes (16%), and asthma (23%). The increases for these conditions were significant in the ARIMA model. There was no significant difference in monthly visits for coronary artery disease or chronic obstructive pulmonary disorder.

**Fig. 3:**
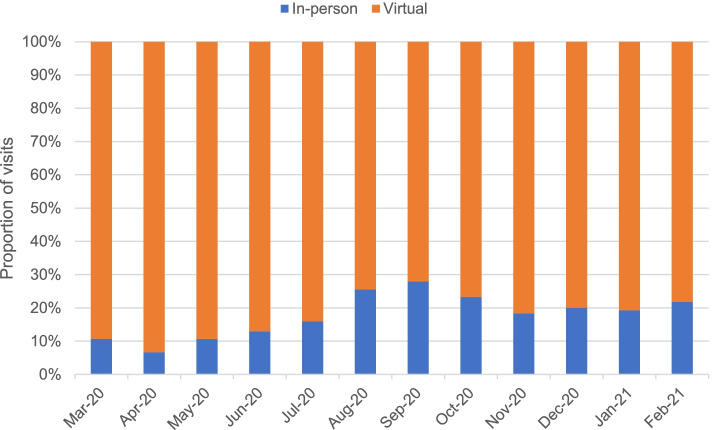
Visit modality proportions by study month in the pandemic period

Figure 3 shows the proportion of visits by visit modality in the pandemic period. The second month of the pandemic (April 2020) saw the lowest monthly proportion of in-person visits at 7% after which in-person visits gradually increased until September 2020, when 28% of monthly visits were in-person. After September 2020, the proportion of in-person visits decreased to 18–23% per month.

### Factors associated with virtual visits in the pandemic period

Table 3 shows patient and visit factors by visit modality after the onset of the pandemic. Patients seen virtually during the pandemic were significantly younger than those seen in-person (mean age 64.9 seen in-person vs. 56.6 seen virtually, *p* <  0.0001). Male patients were more likely to be seen in-person with 20.2% of visits by male patients being in-person compared to 16.7% of visits by female patients (*p* <  0.0001). Patients with an email registered with the hospital were more likely to be seen virtually than those without an email on file (85.7% vs. 76.7%, p <  0.0001).

**Table 3:** Patient and visit characteristics by visit modality in the pandemic period

	In-person visits	Virtual visits	Overall	***p***-value
N	1943	8799	10,742	
Mean age (SD)	64.9 (18.0)	56.6 (18.9)	58.1 (19.0)	< 0.0001
Sex				< 0.0001
Female	1096 (16.7%)	5460 (83.3%)	6556 (100%)	
Male	847 (20.3%)	3339 (79.8%)	4186 (100%)	
Email registered				< 0.0001
Yes	875 (14.2%)	5276 (85.8%)	6151 (100%)	
No	1068 (23.3%)	3523 (76.7%)	4591 (100%)	
Visit type				< 0.0001
New consultation	481 (33.%)	955 (66.5%)	1436 (100%)	
Follow-up	1462 (15.7%)	7844 (84.3%)	9306 (100%)	
Visit diagnosis				< 0.0001
AFib	200 (33.9%)	390 (66.1%)	590 (100%)	
CAD	120 (13.4%)	778 (86.6%)	898 (100%)	
CHF	390 (51.9%)	362 (48.1%)	752 (100%)	
HTN	377 (24.0%)	1194 (76.0%)	1571 (100%)	
DM	231 (4.5%)	4857 (95.5%)	5088 (100%)	
Asthma	469 (35.0%)	871 (65.0%)	1340 (100%)	
COPD	156 (31.0%)	347 (69.0%)	503 (100%)	
Clinic				< 0.0001
AACU	814 (89.9%)	91 (10.1%)	905 (100%)	
AFib clinic	84 (73.0%)	31 (27.0%)	115 (100%)	
Cardiology clinic	311 (11.2%)	2457 (88.8%)	2768 (100%)	
GIM clinic	19 (7.7%)	229 (92.3%)	248 (100%)	
Endocrine clinic	68 (1.8%)	3768 (98.2%)	3836 (100%)	
DM clinic	50 (4.7%)	1106 (95.3%)	1066 (100%)	
Respirology clinic	597 (33.1%)	1207 (66.9%)	1804 (100%)	

*AFib* atrial fibrillation, *CAD* coronary artery disease, *CHF* congestive heart failure, *HTN* hypertension, *DM* diabetes mellitus, *COPD* chronic obstructive pulmonary disorder, *AACU* Acute Ambulatory Care Unit, *GIM* general internal medicine

New consultations were more likely to be seen in-person (33.5%) than follow-up visits (15.7%, p <  0.0001). There were large differences in the proportion of virtual visits by clinic location in the pandemic period. Over 85% of visits to the Diabetes, General Endocrinology, General Internal Medicine, and Cardiology clinics were conducted virtually after the pandemic onset. In contrast, visits to the Acute Ambulatory Care Unit and Atrial Fibrillation clinics were mostly in-person (10.1 and 27.0% virtual visits, respectively). Amongst congestive heart failure visits, 48.1% were seen virtually. Greater than 65% of visits for all other diagnoses were seen virtually. The most common conditions seen virtually were coronary artery disease (86.6%) and diabetes (95.5%).

## Discussion

In this analysis of outpatient visits to internal medicine clinics for ACSCs at a large ambulatory hospital in Ontario, Canada, we found a dramatic shift towards virtual care after the onset of the COVID-19 pandemic, as well as several changes in visit trends for overall visits and by specific visit diagnoses. While new consultations decreased by 10% during the pandemic, this was offset by an increase in follow-up visits leading to an overall 15% increase in visits. Our results showed a transition from primarily in-person visits pre-pandemic to predominantly virtual care during the pandemic. Importantly, there were several factors associated with visit modality in the pandemic period, with patients who were older, men, and without a registered email account more likely to be seen in-person than virtually. There were also significant differences in uptake of virtual care by clinic and visit diagnosis, with less than half of heart failure visits being seen virtually, in contrast to diabetes care which was nearly entirely virtual.

The dramatic shift towards virtual care during the COVID-19 pandemic has been consistently shown in other studies across a number of jurisdictions, which is consistent with our findings [1–5, 10–12, 17–19]. A study of visit trends to Veterans Affairs clinics in the United States during the first 10 weeks of the pandemic showed a decrease in in-person visits by 56% which was partially offset by an increase in telephone and video visits, but overall visits still decreased by 30% [1]. Similarly, other studies from the US and Canada have shown an overall decline in visit numbers after the pandemic [10, 20, 21]. These findings are in contrast to our findings of a significant increase in patient volumes during the pandemic after accounting for prior trends, which was driven by more frequent follow-up visits. The reasons for these differences in findings are unclear and may be due to our study having a longer follow-up period allowing for a correction in visits after an initial drop with the start of the pandemic or due to differences in populations and conditions studied.

A prior study demonstrated that new consultations were postponed while follow-up visits shifted to primarily telemedicine during the pandemic [22], which is consistent with our findings, but the literature on patterns in internal medicine is lacking. We do not have data on the reasons leading to decreased new consultations and increased follow-up visits; however, there are several potential factors. Decreased visits in primary care during the pandemic may have led to decreased referrals as demonstrated in Ontario [10] and other jurisdictions during the first wave of the pandemic [23–25]. Other reported barriers, such as patient concerns about acquiring COVID-19 leading to delay seeking care [26] and decreased access to care [27] may have also impacted new visits. Regarding increased follow-up visits, telephone visits may have facilitated easier access to follow-up visits with a prior study showing that virtual follow-up visits were more efficient [28, 29]. Similarly, both patients and providers have reported feeling telemedicine visits worked best for follow-up visits [30]. Furthermore, with the introduction of billing codes for telephone visits, physicians may be booking more follow-up telephone visits for brief calls that would have previously occurred but not been remunerated. Increased follow-up visits may be due to physicians wanting closer follow-up because they are unable to examine their patients, patients seeking specialist care rather than going to emergency departments for exacerbations of their chronic illness, decreased patient access to primary care [10] leading to more frequent telephone visits with specialists, or physicians feeling more comfortable asking patients for frequent follow-ups because of the convenience of telemedicine [29]. Finally, delays in access to tests and procedures during the pandemic [27] may have led to patients needing to rebook earlier follow-up after completing delayed investigations.

To our knowledge, our study is the first to examine how visits for a range of common internal medicine clinic conditions changed during the pandemic. We found variable uptake of virtual care across different clinics and conditions. Patient visits for CHF, hypertension, and asthma each increased by 20%, whereas atrial fibrillation visits decreased by nearly 20%. We cannot discern the reasons for the variability by condition but there are several potential patient, provider, and system factors that may contribute. Our finding that patients who were older were less likely to use virtual care is consistent with past studies, [12, 21, 31, 32] as well as those showing that older patients are less likely to have internet and are slower to adopt technology [33–35]. Also, sicker or older patients may require more frequent follow-up. For example, CHF patients who are at risk of exacerbation may need closer follow-up to remotely monitor for signs of exacerbation with reduced in-person visits for volume status exams. Similarly, hypertension may lend itself easily to virtual follow-up if patients monitor their blood pressure at home allowing for quick telephone visits to titrate medications [29]. Female patients were more likely to be seen virtually in our study, although findings from other studies on sex differences in virtual care uptake have been mixed [10, 21, 31]. In terms of provider factors, a prior study showed that physicians viewed virtual care as optimally suited for managing conditions that primarily involved counselling and were less reliant on physical exams, and in particular, that hypertension and diabetes were easily managed virtually [29]. Other provider factors that may contribute to variability include familiarity or lack thereof with virtual mediums or supplier-induced demand. Finally, system factors likely contributed to decreased atrial fibrillation visits because of decreased clinic hours and fewer presentations to the emergency department, which is the primary referral mechanism to this clinic.

There are several limitations of our study. First, this was a single centre study of select internal medicine clinics and conditions at an academic hospital under a universal healthcare system in Canada. Our findings may not be generalizable to other conditions or health care systems. Second, visit diagnoses were based on the most responsible diagnoses coded by the visit physician, and for some patients, multiple conditions could have been addressed in a single visit. It is possible that some patients followed for multiple conditions by the same provider (e.g. atrial fibrillation and heart failure) had varying visit diagnoses across different visits despite the same conditions repeatedly being addressed in the same visit. This may contribute to the trends in conditions seen in the pre-pandemic and pandemic periods. However, we expect this to affect a minority of patients and not substantially alter our findings. Third, we were unable to extract data on comorbidities and sociodemographic factors that may be associated with uptake of virtual care such as high disease burden, ethnicity, language, socioeconomic status, and under-housing. Other studies from the US have shown these factors to be associated with telemedicine use [21, 31, 36, 37]; however we were limited to data available within the EMR. Finally, while this study examined trends in outpatient internal medicine visits and uptake of virtual care during the pandemic, we did not examine quality of care, outcomes, or patient satisfaction.

## Conclusions

The COVID-19 pandemic led to a transition in the delivery of healthcare in Ontario from almost entirely in-person visits to over 80% virtual care in a short time period. Virtual care remained the predominant model of care delivery a year after the onset of the pandemic, and there were changes in the types of office visits and visit diagnoses seen during the pandemic. Several patient and visit factors were associated with greater uptake of virtual care. This study contributes to the growing literature on the use of virtual care and the effect of the pandemic on health services delivery. Future research is needed to understand drivers at the patient, provider, and system level led to decreased new consultations and increased follow-up visits. Data from other centres is needed to understand if our observation that patients with certain conditions were more likely to be seen virtually is replicated and understand the drivers behind these differences. Similarly, future research is needed on the equity of virtual care and how certain groups may be disadvantaged by the rapid shift towards virtual care. Finally, given the persistence of virtual care one year from pandemic onset as well as the multiple extensions of the temporary billing codes by the Ontario government, we suspect that virtual care will continue to be utilized in the future as part of the physician’s toolbox to provide quality care. However, this speaks to the importance of future work to understand the impact of virtual care on quality of care, patient outcomes, patient satisfaction, and cost-effectiveness of outpatient care.

## Data Availability

Available on request from Dr. Geetha Mukerji. There were no public or administrative databases used for this project. The sole data source for this project was the Electronic Medical Record at Women’s College Hospital, Epic. The Women’s College Hospital Research Ethics Board approved access.

## Abbreviations

COVID-19: Coronavirus disease 2019
SARS-CoV-2: Severe acute respiratory syndrome coronavirus 2
ASCS: Ambulatory care sensitive condition
WCH: Women’s College Hospital
ARIMA: Autoregressive Integrated Moving Average
